# A Rare Case of Retroperitoneal Immature Teratoma in a Young Adult Male: A Case Report From Eastern Morocco

**DOI:** 10.7759/cureus.66290

**Published:** 2024-08-06

**Authors:** Hind Chibani, Soufia El Ouardani, Fatima Rezzoug, Mohammed Arghal, Rachid Jabi, Karich Nassira, Ouissam Al Jarroudi, Sami Aziz Brahmi, Amal Bennani, Mohammed Bouziane, Said Afqir

**Affiliations:** 1 Medical Oncology, Mohammed VI University Hospital, Oujda, MAR; 2 Medical Oncology, Faculty of Medicine and Pharmacy of Oujda, Mohammed First University, Oujda, MAR; 3 Radiology, Mohammed VI University Hospital, Oujda, MAR; 4 Radiology, Faculty of Medicine and Pharmacy of Oujda, Mohammed First University, Oujda, MAR; 5 Visceral Surgery, Mohammed VI University Hospital, Oujda, MAR; 6 Laboratory of Anatomy, Microsurgery and Surgery Experimental and Medical Simulation (LAMCESM), Faculty of Medicine and Pharmacy of Oujda, Mohammed First University, Oujda, MAR; 7 Pathology, Mohammed VI University Hospital, Oujda, MAR; 8 Pathology, Faculty of Medicine and Pharmacy of Oujda, Mohammed First University, Oujda, MAR

**Keywords:** chemotherapy, residual mass, germ cell tumours (gct), retro-peritoneal, immature teratoma

## Abstract

Teratomas are classified as germ-cell tumors. They occur more frequently in the gonads, but extragonadal localization can also occur. Retroperitoneal teratomas are rare and require multidisciplinary management. We report the case of a 20-year-old patient who presented with an immature retroperitoneal teratoma. The patient initially underwent a retroperitoneal mass resection, which resulted in positive resection margins and a residual mass observed in post-operative imaging, necessitating treatment with platinum-based chemotherapy. The purpose of this publication is to highlight the characteristics of retroperitoneal teratoma, along with diagnostic criteria and treatment approaches.

## Introduction

Germ cell tumors (GCT) are the most prevalent malignancy in young adult men, representing 1% to 2% of all male neoplasms [1]. Teratomas fall under the category of GCT. They are classified as mature or immature, originate from the germ layer, and occur most often in gonads, though they can, in rare cases, be found in extragonadal sites such as the retroperitoneum, which is the second least common site after the mediastinum [2]. Retroperitoneal tumors account for between 0.2% and 0.8% of all tumors, with 6% to 18% of them being teratomas [3]. Clinical symptoms will guide the diagnosis, although they are not specific. A radiological and pathological examination panel is necessary to establish the diagnosis. Serum tumor markers such as alpha-fetoprotein (AFP) and human chorionic gonadotropin (HCG) may be useful in diagnosis and follow-up. Due to the rarity of their pathology, these cases require a multidisciplinary approach and must be discussed by a consultation team. In this study, we report the case of a 20-year-old patient who has an immature retroperitoneal teratoma.

## Case presentation

We present the case of a 20-year-old male patient with no previous medical history who was admitted to the visceral surgery department, suffering from abdominal pain that had evolved for three weeks and worsened three days before admission. The symptoms had been resistant to medical treatment, and he had no other related concerns.

**Figure 1:**
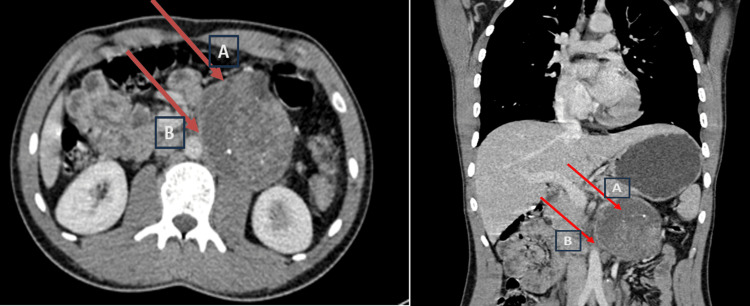
CT abdominal transverse section (left) and coronal section (right) The left retroperitoneal tissue mass contains calcifications and necrotic areas (red arrow A) and has intimate contact with adjacent vascular structures, including the left vein (red arrow B).

A thoracic and abdominopelvic CT scan was performed, which revealed a left retroperitoneal tissue mass containing calcifications and necrotic areas with intimate contact with adjacent vascular structures, including the left vein. These findings were suggestive of a vascular origin, leiomyosarcoma, retroperitoneal sarcoma, or retroperitoneal paraganglioma without any secondary locations (Figure 1).

**Figure 2:**
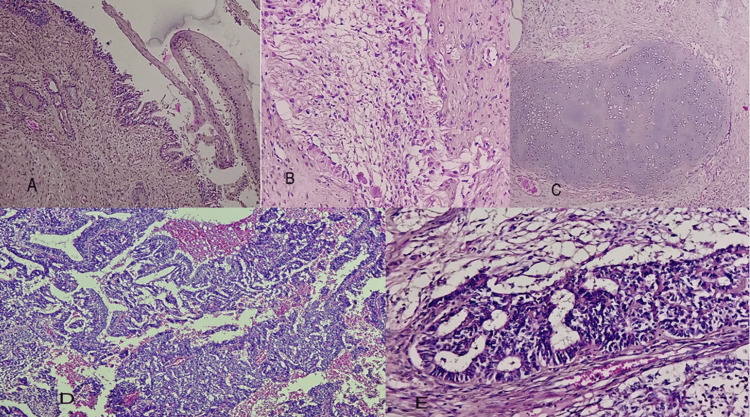
Photomicrographs of immature teratoma (A) Immature teratoma showing bronchial and squamous lining (hematoxylin and eosin x100). (B) Immature teratoma showing nervous tissue (hematoxylin and eosin x200). (C) Immature teratoma with cartilage-like parenchyma (hematoxylin and eosin x200). (D) The immature component of teratoma (hematoxylin and eosin x400). (E) Immature neuroectodermal tissue in the form of tubes (hematoxylin and eosin x200).

The patient underwent a laparotomy with retroperitoneal mass removal. The gross examination and microscopy yielded a pathology report of high-grade immature teratoma with positive resection margins (R1) (Figure 2).

The tumor markers for alpha-fetoprotein (AFP), human chorionic gonadotropin (HCG), and lactate dehydrogenase were negative.

Due to the positive margins, a post-surgery CT scan was performed, which showed a decrease in the size of the left primary latero-iliac retroperitoneal tumor mass that adheres to the residual tumor with left primary iliac lymph nodes.

As a retroperitoneal teratoma was identified, testicular magnetic resonance imaging (MRI) was undertaken. The MRI supported a mixed upper left polar testicular lesion with cystic and calcified components, suspected based on the context. For this reason, the patient underwent a left orchidectomy, which affirmed an epididymal cyst with no evidence of malignancy.

The patient was discussed in a multidisciplinary case consultation. Due to the residual mass, the decision was made to administer three cycles of chemotherapy, consisting of cisplatin 20 mg/m² for 5 days, etoposide 100 mg/m² for 5 days, and bleomycin 30 mg on day 1, day 7, and day 15, every three weeks. The case will be re-evaluated after chemotherapy for potential surgical removal of the residual tumor. Treatment commenced after cryopreservation of sperm.

## Discussion

Extragonadal GCTs can be classified into seminomatous and nonseminomatous types. Embryonal cell carcinoma, choriocarcinoma, yolk sac tumor, teratomas, and mixed GCTs are all subtypes in the nonseminomatous group. Extragonadal GCTs usually occur in midline sites, but their frequency varies with age [4]. Primary retroperitoneal teratomas are observed in both neonates and young adults, as in the case of our patient [5].

The retroperitoneal location occurs early. Teratomas are tumors that contain tissues evolved from omnipotent cells originating from the Hensen’s node. These primordial germ cells, from the 4th week to the 6th week, migrate along the dorsal mesentery to the genital crest, where they turn into gonads. A migration stop or aberrant migration would be responsible for the development of extragonadal tumors. A retroperitoneal teratoma would be an example of migration cessation [6].

The diagnosis of retroperitoneal teratoma requires a clinical, biological, radiological, and pathological correlation.

The presentation is highly variable, ranging from simple abdominal pain to the development of clinical signs related to organ compression, which is largely influenced by the size of the retroperitoneal mass [2].

Imaging is a key tool in diagnosing retroperitoneal tumors. Image findings can help to differentiate between neoplastic and non-neoplastic tumors and to guide the decision, mainly for optimal resection. Different aspects of imaging are present. For instance, immature teratomas are seen on CT and MRI scans, reinforcing mostly solid lumps of variable radiodensities and MRI signal, with foci of fat, calcification, and simple cysts. The infiltration of adjacent structures, especially vascular invasion, is related to worse survival rates [7].

Achieving histopathological examination is the only way to definitively establish the diagnosis. The anatomopathological aspect defined by the WHO 2016 classification recommends classifying teratoma as pre-pubertal or post-pubertal. Although the terms “mature” and “immature” teratoma are often used for classification, they should no longer be used. As both entities can carry a risk of malignancy, this terminology is not clinically relevant and can be misleading. It is necessary to search for somatic-type malignancy without considering the aforementioned classification [8].

During puberty, both the mature and immature components of teratoma can metastasize and are therefore considered malignant [9]. It is worth mentioning that the metastatic potential of teratomas may be influenced by chromosomal aberrations, as many gonadal teratomas have chromosomal anomalies, such as gains in 12p or the aberrant isochromosome 12p (i12p), which suggests a higher risk of malignancy. Additional testing, including cytogenetics or microarray analysis, is not commonly performed at extragonadal sites. Nevertheless, the largest recent case series at an extragonadal anatomic site such as sacrococcygeal localization revealed a negative i12p status, and patient outcomes were favorable [10].

Post-pubertal teratoma is a malignant GCT consisting of diverse tissue types derived from the embryonic leaflets: endoderm, mesoderm, and ectoderm. It can be composed solely of mature, well-differentiated tissues, but it may also contain an immature component. On microscopic examination, all epithelial and mesenchymal tissue types can be found: respiratory or squamous epithelium, pilosebaceous appendages, gastrointestinal or thyroid glands, cartilage, bone, or neuroepithelium are most prevalent [11]. Immunohistochemistry and molecular features, if done, will indicate that keratin is positive, and Sal-like protein 4 (SALL4) may be positive. Also, in the pure presentation, tumor markers such as AFP and beta-HCG are negative [12].

The therapeutic decision should always be discussed in a multidisciplinary team consultation. A complete resection remains the basic treatment [13], where only the resection of the peritoneal mass was considered in old publications [14,15]. However, chemotherapy may be useful for downstaging before surgery [16]. The recommended drug combination, based on the extrapolation of other GCTs, such as ovarian teratoma, is the BEP regimen (bleomycin/cisplatin/etoposide) to avoid recurrence [17]. It is known that teratomas are resistant to chemotherapy; therefore, chemotherapy should be considered in rare instances [18]. Few cases are reported in the literature. In a recent Japanese publication by Ryoichi Maenosono, 32 cases of retroperitoneal teratoma were described, of which 31 patients were treated surgically, while only one patient received chemotherapy [3].

In our case, after identifying a residual mass in close contact with the bifurcation of the abdominal aorta and the primitive iliac artery on the CT scan after surgery, the decision to deliver chemotherapy was made in the hope of decreasing tumor mass before considering an optimal resection.

## Conclusions

Retroperitoneal teratomas are rare, and a synchronous gonadal localization must always be sought. The therapeutic decision must be discussed using a multidisciplinary approach to improve the quality of care. Cryopreservation of sperm should always be proposed to young patients before initiating therapy. Surgery remains the treatment of choice for retroperitoneal teratomas, although chemotherapy and radiotherapy should be considered as part of the multidisciplinary discussion, particularly in the setting of concurrent somatic malignancy within the teratoma.
